# A Systematic Review on Muscle Synergies: From Building Blocks of Motor Behavior to a Neurorehabilitation Tool

**DOI:** 10.1155/2018/3615368

**Published:** 2018-04-22

**Authors:** Rajat Emanuel Singh, Kamran Iqbal, Gannon White, Tarun Edgar Hutchinson

**Affiliations:** ^1^Department of System Engineering, George W. Donaghey College of Engineering and Information Technology, University of Arkansas at Little Rock, Little Rock, AR 72204, USA; ^2^Department of Engineering Technology, George W. Donaghey College of Engineering and Information Technology, University of Arkansas at Little Rock, Little Rock, AR 72204, USA; ^3^School of Counseling, Human Performance and Rehabilitation, University of Arkansas at Little Rock, Little Rock, AR 72204, USA; ^4^Department of Biochemistry and Molecular Biology, College of Medicine, University of Florida, Gainesville, FL 32610, USA

## Abstract

The central nervous system (CNS) is believed to utilize specific predefined modules, called muscle synergies (MS), to accomplish a motor task. Yet questions persist about how the CNS combines these primitives in different ways to suit the task conditions. The MS hypothesis has been a subject of debate as to whether they originate from neural origins or nonneural constraints. In this review article, we present three aspects related to the MS hypothesis: (1) the experimental and computational evidence in support of the existence of MS, (2) algorithmic approaches for extracting them from surface electromyography (EMG) signals, and (3) the possible role of MS as a neurorehabilitation tool. We note that recent advances in computational neuroscience have utilized the MS hypothesis in motor control and learning. Prospective advances in clinical, medical, and engineering sciences and in fields such as robotics and rehabilitation stand to benefit from a more thorough understanding of MS.

## 1. Introduction

The nervous system has two parts: (1) the CNS and (2) the peripheral nervous system. Neural signals from the CNS have repeatedly been observed to activate specific muscle groups during the performance of motor tasks, referred to as motor primitive (MP). How the CNS makes this selection from a seemingly vast pool of such primitives to attain a behavioral goal is a complicated question in the field of motor control. The task is computationally challenging, and for over 50 years, researchers since Bernstein have investigated how the CNS reduces the degrees of freedom by focusing on a smaller set of variables [1]. The evidence in support of the CNS use of motor primitives seems conclusive. Sherrington, Sharrard, and Ferrier and Yeo concluded that after stimulating the spinal cord (SC), the intricate network of nerves resulted in highly coordinated functional synergies in the musculature of dogs, frogs, cats, rabbits, and monkeys [2–4]. The root stimulation of various SC segments in humans resulted in different coordinated muscular reflexes in the lower limb [3]. In this review paper, we will analyze evidence supporting the existence of MS among different species resulting in the modular organization of the CNS. Microstimulation is predominantly used to examine natural motor behavior [5]. The combination of MS/encoded modules produces different natural motor behaviors that are task-dependent (i.e., task-specific MS) or task-independent (shared MS) [6–8].

There is an ongoing debate about the origin of MS whether they have a neural origin or a nonneural origin, that is, whether they are encoded in the CNS or activated because of task constraints. With advances in EMG and functional magnetic resonance imaging (fMRI) data analysis, the current view leans towards the organization of MS as spatiotemporal components in the brain and SC, thus supporting their neural origin. We examine the various techniques and algorithms used for the extraction of MS and present a mathematical model of MS. We also review how the central pattern generators (CPGs) in the absence of peripheral feedback trigger the MS. Further, MS as a physiological marker in stroke patients and its future prospects in neuro-rehabilitation, robotics, and sports science are discussed.

## 2. The Existence of MS

**Figure 1:**
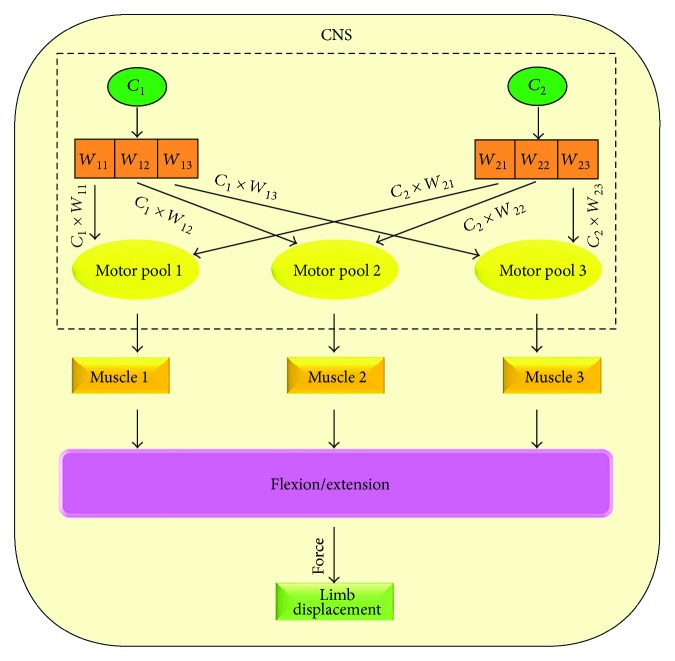
The spatial and temporal pattern of MS encoded in the CNS coactivates the group of muscles. The motor pools from the CNS bring the information as neural command to activate the specific muscles for a particular movement, which results in flexion and extension, generating force and producing movement in space.

The presence of MS in motor tasks has been supported in experimental studies for several decades.  Figure 1 shows how encoded MS/MP in the CNS activates muscles resulting in force production and limb displacement.

### 2.1. Force Fields/Motor Primitives

MS have been associated with the stable force produced by the limbs during natural motor behavior. Maton and Bouisset observed that synergistic groups of muscles produced different external forces during supination or pronation and the external force produced was the sum of the forces of various muscles [9]. Further, the EMG signal was equal to the coefficient times the forces produced by the muscles [9]. It is believed that these forces are present in the CNS and are known as motor primitives (MPs). Giszter et al. during SC microstimulation in frogs concluded the existence of MP [10]. These MPs or convergent force fields (CFFs) present in the SC among vertebrates and invertebrates are the building blocks for complex motor behavior, and by their vector combination, a wide repertoire of behaviors can be generated [10–15]. It has been proposed that the CNS uses these convergent force fields to solve the inverse dynamics problem to reduce the kinematic degrees of freedom [16].

Motor primitives usually develop from a neonatal stage to toddler stage [17]. Some of them are encoded into the spinal cord during skill acquisition. In humans, an internal model of the force field in the CNS is constructed with a given motor task during a robot-guided movement. These newly encoded computational modules represent internal coordinates or muscle/joint coordinates [18, 19]. Huesler further demonstrated synchronization and nonsynchronization of motor units on inter- and intramuscular pairs during the production of force [20]. The motor unit synchronization during precision grip varied along different force and muscle activation levels thus validating the presence of predefined modules. Neuromechanical models provide better understanding of superfluity of muscles and their neural control during a behavioral task [21]. The muscle fibers and motor neurons (MN) together constitute a motor unit (MU); the activation of MU results in muscle activation and contraction [22]. The motor neurons carry this encoded information/motor drives for the coordinated activation of specific groups of muscles for specific tasks; thus, MPs/motor drives are also referred to as MS. McKay and Ting gave more insight into MS as the fundamental blocks for movements using a 3D model of the cat's hind limb [23]. The premotor drives serve as units for muscle coordination. The drives represent MP in the circuitry of the SC, and the unit bursts are the activation command from the central network [24]. Hart and Giszter observed the complex behavior in the brainstem of spinalized frogs, whereby the extracted drives associated with the unit burst from EMG data were focused on a set of muscles [25].

MPs are classified into kinematic (stable correlation between joint angles), dynamic (stable correlation between joint torques), and neuronal, based on previous studies [26, 27]. There is little evidence of kinematic synergies represented in the M1 region (of the motor cortex), but kinematic synergies seem to originate from the MS [28, 29]. The CNS, instead of employing synergies at the kinematic and/or dynamic level, controls them as MS, which are the covariation of correlated, less stable EMG activities [26, 28, 29]. From the current understanding of natural motor behavior, MP can be classified into acquired and adaptive. The acquired MPs are embedded in the genetic design of our nervous system, whereas the adaptive MPs are learned and then get encoded into the spinal circuitry [14, 18, 19, 30, 31]. The changes in the CFFs or MPs cause changes in muscle coordination eliciting different natural motor behaviors [31, 32]. Various techniques like microstimulation, cutaneous stimulation, and N-methyl-D-aspartate (NMDA) iontophoresis were used to understand the relation between MP and motor behavior [11, 32–35]. The presence of MP is supported by recent developments in the field of computational neuroscience.

### 2.2. Combination of Motor Modules

We have already discussed the presence of CFFs or MPs in the spinal circuitry, which gave us an idea that endpoint forces are dependent on coactivation of muscles. The discussion will now focus on how these primitives are organized in the spinal circuitry and how they are combined to produce various movements. Georgopoulos et al. observed that during reaching movements, the cellular discharge was higher in the rhesus macaque motor cortex when the movement was in the desired direction [36]. It was concluded that the preferred location of the limb in a 3D space is achieved by the vector sum of motor cortex's cellular discharge (population coding). Mussa-Ivaldi et al. investigated simultaneous stimulation of two sites in the spinal cord resulting in the endpoint forces being equivalent to vector summation of each site after costimulating them individually in spinalized frogs [37]. The outcome was the same in cats during intracortical microstimulation (ICMS) of the primary motor cortex [38]. Tresch et al. and Kargo and Giszter also found the summation of primitives in vertebrates using ICMS and cutaneous stimulation [35, 39]. Lemay et al. used EMG to compute the forces at the muscular level and mechanically at the ankle in hind limbs of spinalized frogs [40]. During intraspinal electrical stimulation in the spinalized frog, the endpoint forces were the vector summation of each stimulated site. Capaday and van Vreeswijk demonstrated a mechanism in which the motor output is a linear summation without being affected by the presence of nonlinearity (intracortical and intraspinal nonlinearity) in the neural circuits [41]. It was concluded that such nonlinear neural circuit elements do not affect the linear summation of the motor output.

It is interesting that in vertebrates, summation of the motor unit action potentials (MUAPs) is observed using glutamate iontophoresis or electrical stimulation of neurons [38, 42], which is very much similar to MP summation during stimulations of the SC. Electromyographic signals being an algebraic summation of MUAPs are extensively utilized for the extraction of movement primitives or MS by employing decomposition algorithms [40–42]. This understanding leads us to the neural basis of MS.

### 2.3. Neuromechanical Origin of MS

The modular organization of MS in the SC is linked to their neural origin [7, 12, 13, 17, 25, 43, 44]. The feasible force fields with synergies leading to a volumetric reduction of the forces offer dimensional control criteria [45, 46]. Thus, a low-dimensional spatiotemporal structure can explain the muscle activation pattern and the neural origin [6, 23, 43, 45, 47–50]. The low-dimensional space was also consistent between stroke patients and normal patients and within the stroke patient's affected and unaffected sites leading to the speculation of the neural emergence of MS [51–53]. Focal intraspinal N-methyl-D-aspartate (NMDA) also supports the existence of encoded MS in the CNS [32].

d'Avella et al. extracted MS during different natural behaviors in frogs [6]. Their results suggest that a small number of components were sufficient to explain the high-dimensional space vector of the time-varying muscle pattern. The low-dimensional space observed was independent of the task performed as synergies for kicking in frogs were similar between different motor behaviors and hence called shared MS. The lack of similarities in the synergies during different behaviors is due to task-dependent synergies. Such low dimensionality is dependent on task constraints. d'Avella and Bizzi extracted synchronous and time-varying synergies in frogs and found that most synergies were shared, but synergies associated with biomechanical constraints also existed [7].

The synergies extracted during postural tasks in humans had a low-dimensional space [48]. That study also suggested that MS from the CNS were changed during postural adjustment and the number of extracted synergies remained consistent suggesting the neural origin of MS in cats, frogs, humans, and primates [45, 48, 54–58]. The intrasubject consistency of MS was observed during different tasks, thus concluding modulation of MS recruitment instead of its structure [59]. However, dorsal root transection on frogs altered the temporal pattern and amplitude of activation coefficients (structure) of shared synergies. The fine-tuning of the synergies was not consistent within the frogs because of different musculatures [60]. A low-dimensional space is related to neural control and reduction in degrees of freedom [6, 23, 43, 61–63]. Valero-Cuevas et al. provided evidence against the lack of dimensionality, thus arguing against the concept of MS by employing a simple task using intramuscular EMG in the index finger [50]. The argument against the synergy hypothesis is that they only exist during open-loop tasks. However, the claim that MS are hard to reveal in the closed-loop tasks because such tasks are not rich in behavior, is not well supported since MS are well suited with open-loop and closed-loop tasks and can be implemented as an optimal feedback controller. A slight variation in the task should display their variability [7, 45, 50].

Kutch and Valero-Cuevas, using cadaveric and computational models, showed that the biomechanics of the limbs required change in muscle-tendon length to a low dimensionality in different movements. Each muscle group varied the length change individually leading to the emergence of nonneural coupling between muscles and the presence of dimensionality reduction constraints during isometric force production in different directions [64]. The muscles were individually coordinated instead of being grouped together in this experiment. A low-dimensional space originated directly from motor commands (feed-forward MS), biomechanical constraints, and the individual response of each muscle (feed-backward MS) in the experiment. The endpoint forces for feed-forward MS were static as the dimensional space emerged directly from motor commands, contradicting the fact that variation end-point forces with different tasks is commonly observed on a cellular and physical level (cortical discharge) [15, 36, 65–68]. This concludes that the CNS does not need to control a group of muscles to observe EMG signal of low dimensionality. De Groote et al. using a musculoskeletal model and minimized muscle effort found that MS existence is dependent on the task constraints rather than on neural control [69].

Criticism of the synergy hypothesis because of lack of dimensionality could be due to the data processing approach and noise. These factors make it harder to reveal the reduced dimensionality in EMG signals [50, 69]. Bizzi and Cheung also advocated for the neural origin of MS by discussing the dimensional reduction of the subspace with task constraints [17]. For isometric force production in lower limbs, seven synergies were required but the dimensionality reduced further to 4-5 synergies during locomotion [17, 64, 70]. The dimensional space was lower than expected from the task constraint, as it is the neural signals that limit motor output required for the task [21]. Countering Kutch's experimental model, they added that the organization of muscles around a joint results in muscle coupling as it produces regularities in EMG [17, 64]. The question raised by Bizzi and Cheung on the lower limb producing isometric force with similar dimensionality can be understood from Hagio and Kouzaki [17, 43]. The latter studies provide strong evidence for the neural basis of MS in the lower limb during isometric force production in a 3D space. There are many factors that affect the dimensional subspace: the data processing approach and noise factor [69], smoothing or regularities in the EMG signal [71], and the change in muscle-tendon length synchronously [17]. During tasks involving 52 postures and recorded EMG from intrinsic and extrinsic hand muscles, Weiss and Flanders concluded that the MUs from the CNS were linked to coactivation of multiple muscles [72].

**Figure 2:**
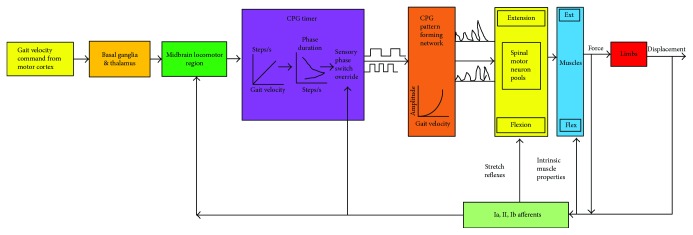
CPG timer circuit in the absence of peripheral feedback coordinates the movements. The figure shows the neuromechanical tuning [78, 79]. The primary motor cortex region dictates the movement via the basal ganglia and thalamus to the MBLR which is a part of the brainstem where spatial–temporal patterns are encoded. Force and displacement are sensed by the muscle spindle and the Golgi sensory receptor through a feedback.

With these evidences, it would be correct to infer that MS have neural basis. The feedback from the peripheral nervous system assists to acquire MP into the spinal circuitry for a certain task. The stored MP will assist the CNS to adjust biomechanics of the limbs to accomplish the task [73]. In short, the neural control in most cases overcomes the passive dynamics but in some instances utilizes the passive dynamics to achieve the task [21]. In the absence of sensory feedback, a CPG reduces the dimensionality of locomotion [74]. The CPG generates the MS with variation of the task by the process of neuromechanical tuning in rhythmic movements, and thus it is more appropriate to say that MS have a neuromechanical origin [12, 21, 61, 73–80]. This process is clearly defined in Figure 2.

## 3. Brain and MS

Recent research has often employed brain fMRI to study MS. When performing a task, the primary motor cortex (M1) region exhibits changes on a cellular level by a high neuronal discharge rate in a preferred direction [15, 36, 66]. In M1, the axons of Betz cells and cortical neurons descend from the brain to the SC forming corticospinal pathways. Cash and Yuste demonstrated linear summation of excitatory inputs by pyramidal neurons (Betz cells) in the hippocampus (i.e., sliced biopsy) of rats [81]. Like MPs, linear arithmetic of neurons provides independent processing of multiple channels without disruption of information [41, 81]. The M1 is subdivided into the rostral and caudal regions in higher primates including humans. The former mediates motoneuronal activity in the SC, and the latter is known for higher skill acquisition by skipping the spinal circuitry [82]. Cherian implanted electrode arrays in the M1 region of rhesus macaques. The outcome was that under a force field, the neuronal discharge was synchronous with muscle dynamics and M1 did not account for motor learning directly. Law et al.'s research with implanted microelectrode arrays in rhesus macaques while performing a task showed that the extra group of neurons was comodulated from M1, and such flexibility of M1 resulted in enhanced motor skills [83]. The MS encoded into the spinal circuitry are updated by the cortical regions through corticospinal pathways pertaining to specific tasks. The flexibility of these task-specific MS is restrained by the unequal combination of muscle fields [84]. In rhesus macaques, electrical microstimulation of the motor cortex generates synergistic muscular activity for multiple degrees of movements [33, 34]. Decomposition algorithms were used on muscle and motor cortex data acquired after microstimulation of the cortical region while performing different tasks. The neuronal firing pattern decomposed and corresponded to the same dimensionality as that from the muscle recordings, which concluded the presence of spatiotemporal synergies in the brain [34].

Using transcranial magnetic stimulation (TMS), it was found that the human cortical region is associated with the activation of different synergistic groups of muscles in the lower portion and this was validated with EMG and fMRI recordings [85]. The cortical regions are the center for muscle coactivation or MS pertinent to functional tasks [86, 87]. Optimal coordinated movement is caused by the convergence and divergence of the corticospinal system with central neuromotor noise. The organization of MS in the primate motor cortical region has been studied, and synergies in this area are designated as discrete spatiotemporal synergies [34]. In phylogenetically advanced species, while grasping, instead of synergetic control of muscles, a more fractionated control is superimposed [88]. The fractionated control is due to the new cortico-motor neuronal pathways which bypass the spinal circuitry. The new M1 cells in higher primates are associated with these fractionated control pathways which are also important for skill acquisition [82, 88]. This novel approach of the CNS is the foundation for the development of prosthetic arms.

The feedback mechanism is important for movement, as in grasping MS are modulated with respect to the variation in the shape of object among monkeys [56, 89]. In the primary motor cortex, M1 dictates reaching and grasping objects as a single movement separated more in time than in space [15, 54, 90]. However, mammals with transected SC produced rhythmic movements revealing the presence of CPGs in the CNS. Brown conducted an experiment with afferented and deafferented cats and found similar MS in both groups of animals [80]. The CPG activates the muscle groups that generate rhythmic patterns in vertebrates without sensory feedback [75, 76, 80, 91]. In the absence of a sensory feedback or during rhythmic movements like running, modulation in CPG by neuromodulators results in the modulation of the timing, duration, and magnitude of MS [54, 70, 74, 91, 92].

Neural signals are transmitted from the basal ganglia and thalamus to the midbrain locomotor region (MBLR) and to the SC for CPG generation [54, 76] (Figure 2). Prochazka and colleagues [78, 79] and Drew et al. [86] concluded that the motor commands pertaining to movement pace originate from the cortex region, channeled towards the SC via the MBLR. It drives the CPG timer in the SC to produce cadences with the flexor and extensor phase duration. During the change in velocity of the rhythmic movements, the CPG pattern formation network driven by the motor commands modulates the activation of the muscles as per square law relationship [78]. Hence, CPG phase durations and the muscle forces match without the presence of a sensory feedback with biomechanically changing events. Overduin et al. using ICMS provided a better insight into the location of MS [5]. The spatiotemporal patterns rather than being encoded into the cortex region were present in the deeper part of the brain (brainstem or SC) [5]. The alpha-motor neurons in the brainstem and spinal cord were activated during kinetic and kinematic gait events that map spatiotemporal patterns [93]. We could infer that the M1 region dictates the movement through alpha-motor neurons, Betz cells, and other cortical neurons by triggering the MS in the SC or brainstem.

## 4. Algorithms for Extracting MS

Muscle synergies as a linear combination are usually extracted using matrix factorization algorithms like the independent component analysis (ICA), nonnegative matrix factorization (NNMF), and factor analysis (FA) [94]. The application of dimensional reduction algorithms on EMG data is important to observe voluntary or nonvoluntary movements because the planning of movements happens in a low-dimensional space. The mathematical model of MS is classified into time-varying synergies (spatiotemporal synergies) and time-invariant synergies (spatially fixed or synchronous and temporal pattern) [95, 96]. The spatial and temporal synergies are elicited individually by the CNS [49]. The nonnegative matrix factorization multiplicative method is commonly used to extract the MS. Being an optimization algorithm, the linear decomposition minimizes the reconstruction error [6]. It is also important to determine which type of MS needs to be extracted from the EMG. The point process statistics method was used by Hart and Giszter to distinguish between time-variant and time-invariant MS prior to extracting them from the EMG pulse timing or onset timing [97].

Equation (1) gives a mathematical model of MS: here, *E* is the EMG signal with *n* sensors (or muscles) and a total of *m* samples, **W** is the synergy matrix with reduced dimensionality, *s* is the number of components or dimensions to be extracted from the EMG, *C* includes coefficients of neural command vectors and *e* is the residual error.

(1) $$\begin{array}{c} E_{n\times m}=W_{n\times s}\times C_{s\times m}+e. \end{array}$$

(a) Time-variant synergies:
  (2) $$\begin{array}{c} E\left(t\right)=\overset{N}{\underset{i=1}{\sum }}W_{i}C_{i}\left(t-t_{i}\right), \end{array}$$

where *N* is the number of synergies and *E*(*t*) is the activation of muscles at time *t*. The individual synergy vectors are shifted in amplitude and scaled in time by the activation coefficients [95].

(b) Time-invariant synergies:
  (3) $$\begin{array}{c} E\left(t\right)=\overset{N}{\underset{i=1}{\sum }}W_{i}C_{i}\left(t\right). \end{array}$$

The spatial patterns W are the task-dependent control inputs whereas the activation coefficients C are the task -independent predefined modules [95].

Artificial neural networks (ANN) have been similarly employed to extract MS [98]. The spatial and temporal patterns from EMG were estimated by the algorithms with no unique solution [99]. The performance of PCA is not laudable when compared with that of other algorithms [45, 50, 94, 100]. Tresch et al. compared the performance of different algorithms [94] using a simulated and recorded (EMG) data set with nonnegative values. The performance of each algorithm is listed in Table 1.

Principal component analysis sometimes gives negative values in the synergy subspace, which represents the inhibitory response of the spinal circuitry [94]. Krishnamoorthy et al. used an uncontrolled manifold approach (UCM) hypothesis to extract postural MS [48]. An autoencoder performed better as a synergy extractor than other algorithms while reconstructing the EMG data; it extends its performance by providing an agonistic and antagonistic relationship between muscles [98]. Principal component analysis also provides such relations but does not offer good reconstruction efficiency [98]. Algorithms like NNMF and probabilistic independent component analysis (pICA) that utilize the hypothesis of muscle synergies performed with higher classification accuracy to distinguish between the single and multiple degrees of freedoms of upper and lower extremities [101, 102]. Electromyographic signals being corrupted by signal-dependent noise could be the reason that these algorithms conveyed better classification accuracy since NNMF and pICA execute well with signal-dependent noise [25, 101–104].

The dimensionality of control commands (neural commands from the CNS) is not exactly the same as the elements of the state space (musculoskeletal structure); therefore, the exact number of synergies needs to be determined prior to extracting them using different computational algorithms [48]. The MS analysis is dependent on many variables including muscles included, algorithms, EMG normalization method, constant or varying synergy vector (SV), output vector normalization method, and synergy comparison method [105].

Bartlett's test, Akaike information criteria, Bayesian information criteria, Laplacian information criteria, and Likelihood ratio test can all be used to identify the correct number of synergies for Gaussian noise-corrupted data but fail to do so for signal-dependent noise. For signal-dependent noise, an ad hoc procedure based on log-likelihood curves may identify the correct number of synergies [94]. For estimating the count of MS in comparison to the ad hoc procedure, *R*-squared curve or VAF (variance accounted for) curve gives a more accurate estimate. By definition, VAF and *R*-squared are similar (1 − sum of squared errors/total sum of errors), but in standard Pearson correlation, the total sum of squared errors for VAF is with respect to zero not the same with that for *R*-squared which is related to the mean.

### 4.1. Procedure (NNMF Multiplicative Update Method)

**Figure 3:**
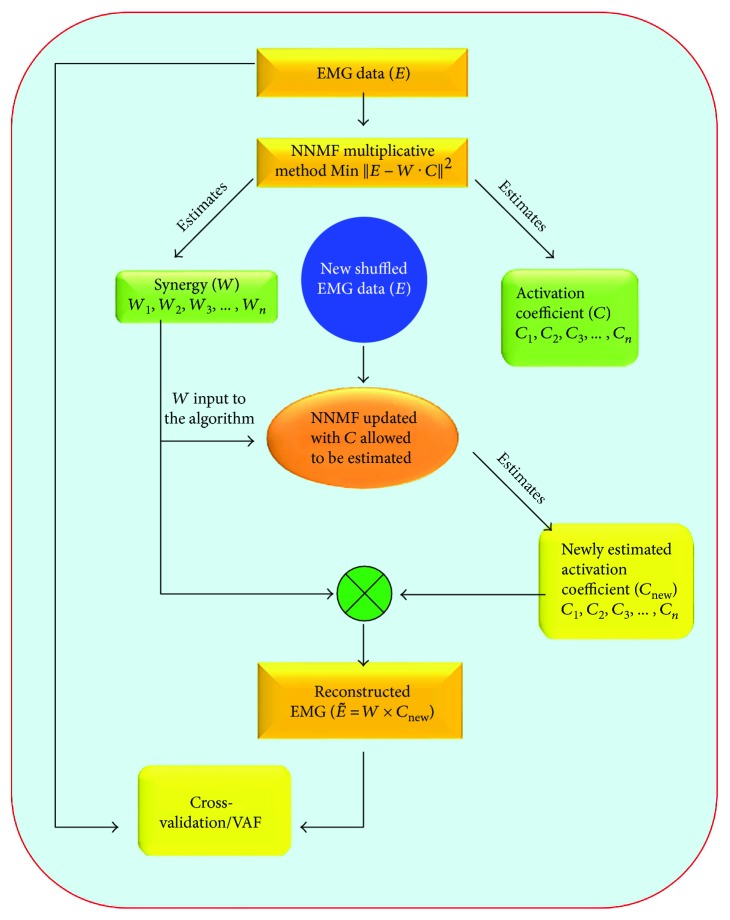
Procedure to extract MS from the NNMF multiplicative method. Synergies (*W*) were extracted from raw EMG data. EMG data is then shuffled and fed again to the algorithm with *W* as fixed synergy, and activation coefficient (*C*) is allowed to be estimated. The EMG signal was reconstructed with *W* and newly estimated activation coefficient (*C*_new_). Cross-validation is performed between the original (*E*) and the reconstructed EMG data.

For determining the applicable MS from raw unshuffled EMG data, *N* synergies (1–10) were extracted from EMG data set with *n* muscle and *m* samples (Figure 3). The algorithm estimates *W* and *C*, the EMG data is shuffled and fed to the algorithm again with earlier extracted synergies as fixed synergies, and the activation coefficient (*C*) is kept unfixed and allowed to be estimated. The EMG data is reconstructed again by multiplying the newly estimated activation coefficient vector and fixed synergy vector *E* = **W** × **C**. For each synergy point, the data is reconstructed; then, *R*-squared or VAF curve is plotted against the *N* number of synergies (1–10) using the expression (1 − sum of squared errors/sum of total squared errors).

(4) $$\begin{array}{c} SSE=\sum \sum {\left(E_{nm}-{\hat{E}}_{nm}\right)}^{2},\\ SST=\sum \sum {\left(E_{nm}-{\bar{E}}_{nm}\right)}^{2}. \end{array}$$

Here, *n* is the number of muscles and *m* is the number of samples. There are four main ways to calculate the number of MS based on the VAF curve, which are as follows:

(1) Best linear fit (BLF) method: Cheung presented that moving along with a greater number of synergies or components on the *x*-axis of the graph reduces the mean square error and the curve becomes a straight line. The point at which it attains the plateau is chosen for the correct number of synergies [60].

(2) Knee point (KP) method: Cheung et al. presented another method in which the point or the number is chosen where the curve has an increase of smaller than 75% [51].

(3) Elbow method: Tresch et al. suggested the point at which the change in the slope of curves is maximum [94].

(4) Threshold method: Torres-Oviedo et al. used a threshold basis of 0.9 (90%) on the VAF curve to find the number of synergies for extraction [58].

### 4.2. Automated Task Decoding Method

This method uses different decoding algorithms (linear discriminant analysis (LDA), quadratic discriminant analysis (QDA), naive Bayesian (NB) algorithm, and K-NN clustering) and statistical testing. The number of synergies is chosen at a point where there is no statistical significance after adding more synergies to the decoding parameters of the algorithm. The statistical significance is computed between the decoding parameters of *N*, *N-1*, and *N*th synergy which are pseudo-randomly shuffled 100 times [103].

There is no perfect algorithm for MS estimation because with biomechanical constraints, the accuracy of the algorithms gets reduced [100]. In the classical VAF method, the variance causes difficulty in choosing the exact number of synergies. Delis et al. presented a more accurate method to extract a smaller set of synergies from the data [103]. In this method, the performance of synergy computation is dependent on the algorithm type as LDA performed faster and more precise using the dataset in their study.

## 5. MS as a Physiological Marker for Neurorehabilitation

### 5.1. Stroke

Muscle synergy structures based on motor control outputs are beneficial for recognizing alterations in the brain for various motor tasks. Thus, MS prove to be important in the field of human locomotion and neurorehabilitation as motor impairments can be understood by means of the patterns or structures of MS [53, 106–108]. Cheung in their study in ischemic stroke patients with affected and unaffected arm found MS similarities in both arms with respect to different lesion sizes and locations in frontal cortical areas. This led to the conclusion that the cortical signals activate the muscles at both sites in a similar way [60]. However, due to cortical lesions, altered activations of muscles occur resulting in deficit motor performance. This altered coordination can be emphasized for stroke recovery. Alteration in the MS is dependent on the severity of impairment [107, 109]. Recent studies provided additional factors which affect the structure of MS or possibly the similarities in MS. In most cases, alteration in MS is observed, but the results by Cheung showed that preserved synergies could be due to hand movement with the intact sensorimotor cortex [60]. Merged synergies are associated with the abnormal coupling of joints in chronic stroke patients during different movements [52, 110]. These abnormal synergies, instead of being eliminated, can be augmented through robot-assisted therapy [53, 108, 111, 112].

We have emphasized the impact of MS with respect to the level of impairment or cortical lesion, but the alteration in MS can also result with time among stroke patients. There are three main stages of stroke categorized on the basis of duration by computed tomography (CT).

#### 5.1.1. Subacute Stroke

The time period for the stroke is from 48 hours to several weeks. The upper-limb MS in such stroke is very similar to that in healthy patients, as the previous study also revealed, but the neural drives or activation coefficients displayed alteration because of the presence of a cortical lesion or damage in the cerebral hemisphere [108]. Hashiguchi et al.'s study on subacute stroke patients revealed both merging and fractionation of MS in the lower limb during gait; however, unlike addition of more synergies for good motor performance [52], the merging of synergies resulted in poor muscle coordination. It is possible that because of the short time span, new synergies were not added to the subspace for flexible control of the limb by stroke patients.

#### 5.1.2. Acute Stroke

The time period is very short which is less than 24–48 hours. Any voluntary movement is less likely.

#### 5.1.3. Chronic Stroke

The time period ranges from months to years. In chronic stroke, fractionated and merged MS were observed [109]. In such condition, MS are not usually conserved as it appeared in earlier studies. The alteration in proximal MS is more distinct in severe cases than in mild and moderate cases, although some synergies in mild, moderate, and severe stroke were conserved in elbow flexors and extensors but varied in shoulder muscles. The conserved, merged, and fractioned synergies exist together in stroke patients [52, 109]. The difference in the similarity of the MS could be due to the neuro-anatomical sites or intact sensorimotor cortex [106, 113]. With the intact sensorimotor cortex, MS similarity is poor with the newly generated MS. In contrast to this without the intact sensorimotor cortex, MS similarity is higher in chronic stroke [106]. Mcmorland et al. further added that the degree of similarity and preservation of synergies among individuals with intact sensorimotor cortex was positively associated with hand function [113]. Godlove found that MS after stroke are related to perilesional high gamma observded in the electrocorticography (ECoG) signals [114].

### 5.2. Fugl-Meyer Assessment

The studies discussed so far show consistency to the Brunnstrom approach that includes stages of motor recovery. The MS count remained static in pain with altered spatiotemporal patterns in the upper limb [115]. In stage 3, the pain is high because of muscle stiffness and it can be inferred that with decreasing spasticity stages, emergence of new MS or augmentation of affected MS for rehabilitation is likely to be achieved successfully. The following stages were implicated: (1) flaccid paralysis: there are no reflexes; (2) appearance of spasticity: basic MS start appearing and abnormal movement of limbs will be present; (3) increased spasticity: there is increased stiffness of the muscles and voluntary movements can be attained, but motor control is absent; training of muscles or MS in this stage can lead to preliminary recovery; (4) decreased spasticity: motor control starts appearing and training should be continued; (5) complex movement combination: here, the MS patterns get more coordinated and complex movement can be attained by the limbs; (6) disappearance of spasticity: with the disappearance of spasticity, increasing motor control is achieved; difficulty during rapid complex movements remains and MS are more coordinated; and (7) normal functioning: normal functioning of the limbs as optimal control is attained.

Based on the above approach, Brunnstrom and Fugl-Meyer et al. developed the scoring system for the quantitative clinical assessment of five domains for rehabilitation [116, 117]. The five domains of the assessment are the motor function, sensory function, joint pain, joint motion, and balance [116, 117].

### 5.3. Rehabilitation

In patients with a neurological disorder, the imbalance of muscle coordination is compensated by other muscle groups. For example, in chronic stroke, trunk synergies are compensatory to overcome aberrant coupling in the arm for better control [112, 118]. Similar compensatory synergies have been observed in lower limbs during gait [119]. These compensatory synergies inhibit the recovery process of specific MS as it becomes hard to train the specific muscle groups to recover. Thus, specific muscle synergies should be trained and any compensatory muscle synergies should be inhibited during the training period depeding on the level of impairment among patients [112]. In contrast, studies also suggest that this compensatory behavior should not be ignored but rather be utilized for better poststroke recovery [119, 120].

Fractionated MS are observed in poststroke individuals after several years. It can become the flexible control strategy for movement as synergies are added. The formation of new synergies, which were evident in chronic stroke, can be useful for neurorehabilitation as these synergies were adapted for improved controllability of limbs [51, 121]. A minimum of six-week repetitive training can induce changes in the white matter to build new or augment existing synergies [53, 106, 121]. In the lower limb, while standing, stroke-affected patients and healthy individuals exhibited common synergies, since the stroke patients shifted the center of mass to maintain balance and their MS were similar to those of a healthy person [48, 122, 123]. In most studies, NNMF (multiplicative method) is used to study the synergies among stroke patients [51, 52, 107, 109]. Different methodological techniques for synergy extraction can provide better information on a trial-by-trial basis or on an individual basis for better recovery from stroke.

## 6. MS Application in Sports

Muscle synergies have been analyzed thus far during normal physical activities, but during peak physical activities, the role of MS with respect to the task is also of great interest. The distribution of relative weight of the muscles in the synergy space helps in understanding the movements. Modulating compensatory synergies that minimizes the chances of injury by specific training is a better solution in sports rehabilitation and performance. Matsunaga et al. analyzed the MS before and after 10 minutes of running [124]. Similar to previous studies, the number of MS was consistent. The first three modules or MS were similar, but the fourth MS showed the activation of muscles around the pelvis region moving from the trunk region. This could be the reason that during foot strike, we are more prone to injury as the postural control has been shifted from the trunk to the lower limb.

Some sports like gymnastics require high postural stability. Trunk muscles play a vital role in human postural control [112, 118, 124]. Among gymnasts, while performing a giant swing, three synergies were identified [55]. The first two synergies were consistent between subjects, but the third synergy displayed variability. Frère and Hug concluded that the variability in the third synergy could be related to the lower-level neural control rather than to the biomechanical constraints. Here, the synergies associated with the trunk, arms, and shoulder muscles cooperate to limit further the extension of the shoulder joint [55], thus reducing the chances of injury. A recent research showed that after five weeks of strength training (bench press), there was an intrasubject variability of MS [125], whereas those who had not trained themselves and continued with their daily routine showed no such variability [125].

Shaharudin et al. analyzed a group of untrained individuals performing rowing movements on slide and fixed ergometers [126]. The results were consistent with those of previous studies. Muscle synergies were modulated, but their structure remained the same [59, 126]. The CNS had distributed the weights of the muscles differently for rowing on a slide ergometer (leg muscles) and a fixed ergometer (back muscles) to minimize injuries. We can say that the nervous system continuously makes adjustments to optimize the selection of MS that are best suited for the specific movement. It is reasonable to assume that this optimization of the MS selection process is based on identifying and utilizing the most economical MS profile for the movement and one that reduces the chances of injury. Barroso's research on MS stated that functional motor impairment (spinal cord injury) quantitative assessment was performed well while cycling. Muscle synergy modulation plays a vital role in sports performance and, in the future, can become a vital tool in recovery from injury [55, 124, 126].

## 7. MS Application in Robotics

In this section, we will discuss the application of MS in robotics. A low-dimensional controller controls the dynamics of the limbs without losing the performance. A number of studies have utilized the hypothesis of MS to build artificial limbs [101, 102, 127]. Berniker et al. built a simple controller with MS and a low-dimensional model whose performance is close to a full-dimensional controller [61]. Alessandro et al. in his review presented the principal foundation of using the hypothesis of MS in robotics [95]. The synergies extracted from the task are combined to form the motor signal for a newtask and were tested further based on the observed task [95]. Including the dynamics of the musculoskeletal model can help better approximate muscular activities. Rasool et al. examined the state space modeling, used the MS matrix extracted from EMG data as an observation matrix, and included the dynamical model of the upper extremity [102, 128]. The neural drive was further estimated using an updated state-constrained Kalman filter that has a synergy matrix and the upper-extremity dynamic matrix, which can be used to control upper-extremity myoelectric prostheses [102, 128]. Afzal et al. did similar work on the lower limb during overground movements [101]. Direct and pure kinematic modeling of the synergy patterns could result in inconsistency in grasping [129]. Pisa/Italian Institute of Technology modeled a robotic hand with the concept of translating soft synergies into adaptive synergies to solve this issue. To implement such model, underactuated hands with designed ligaments and innovative joints were used. The underactuated hand's parameters were selected to mimic the given synergies for consistent grasping [127, 129]. There are currently many improvements under way, and dynamical systems have been developed for movements that are more complex. This will lead to the addition of more flexible movements in robots with higher controllability.

## 8. Conclusions

We have presented recent research results related to MS in motor control, neurorehabilitation, robotics, and sports science. This review paper concludes that the modular organization of MS in the CNS and their combination lead to a variety of natural motor behaviors. These predefined encoded primitives reduce the dimensionality of the behavior for better task control [23, 45, 53, 54, 57, 58, 61, 130]. The concept of MS is still under study with critical and unbiased views towards it [13, 17, 59, 64, 104, 131]. We have postulated that the MS are represented as spatiotemporal synergies in the CNS and are triggered individually as spatial and temporal patterns by the CNS [43, 47, 49, 88]. The linear decomposition algorithms (PCA, NNMF, and ICA) are predominantly used to extract spatiotemporal, temporal, and spatial synergies from EMG. In the fields of neuroscience and robotics, the MS hypothesis has proven to be efficient for motor control of the limbs by reducing the degrees of freedom [101, 102, 127, 132].

Temporal synergies are crucial in understanding the neural basis of MS [43]. The case for the neural origin of MS is still a question of debate, but the evidence shows a strong inclination towards its neural-physiological origin as well as spatiotemporal pattern representation in the brain and the SC. We have also concluded that the alteration in MS is dependent on the site of lesions, the severity of impairment, the stage of stroke, and, to a certain extent, the complexity of the task performed by the patients. As MS can be preserved, fractionated, and merged among stroke patients, this makes it a physiological marker for neurorehabilitation. The emergence of new MS and augmentation with robot-assisted therapy and sports and exercise therapy provide a relation to changes in the white matter and neuroplasticity [133]. The MS can also be used to assess the distribution of the muscle weights during movements that are more vigorous. The abnormal shifting of the activation of the muscles can be observed from MS patterns and hence can help us in reducing the chances of injury [124].

Besides stroke, other neurological disorders like cerebral palsy, dystonia, and spinal injury have been investigated by the hypothesis of MS, which allow flexibility in neurorehabilitation or diagnosis in these and other disorders [8, 134–138]. The association of MS and CPG provides a new approach for myoelectric prostheses among arm and leg amputees by reducing the degrees of freedom [101, 102]. Instead of considering MS as implemented in software, there are some applications in robotics where a hardware model has been built around an MS concept [127, 129]. Most of the MS studies utilize different algorithms, and to this date, no single approach to optimally process the EMG signal and extract MS has been identified. Studies have revealed that different algorithms responded differently to noise in the EMG source and were likely to affect the accuracy or precision of the results. There is still considerable room for further research related to MS in the field of neuroscience, robotics, and sports.

## Figures and Tables

**Table 1:** The spectrum of performance of different algorithms for synergy estimation from the EMG signal affected with Gaussian noise and signal-dependent noise performance of algorithm in the identification of the subspace and activation coefficients [94].

Performance	Gaussian variance noise (synergy estimation)	Signal-dependent noise (synergy estimation)	Identifying subspace	Activation coefficient
PCA	Low	Low	High	Intermediate
ICA	High	Intermediate	Low	Low
FA	High	Intermediate	High	High
NNMF	Intermediate	Intermediate	Intermediate	Intermediate
ICAPCA	High	High	High	High
pICA	High	High	High	High

## References

[1] Bernstein N. (1967). *The Co-Ordination and Regulation of Movements*.

[2] Sherrington C. S. (1892). Notes on the arrangement of some motor fibres in the lumbo-sacral plexus. *The Journal of Physiology*.

[3] Sharrard W. J. W. (1964). The segmental innervation of the lower limb muscles in man: Arris and Gale lecture delivered at the Royal College of Surgeons of England. *Annals of The Royal College of Surgeons*.

[4] Ferrier D., Yeo G. F. (1881). The functional relations of the motor roots of the brachial and lumbo-sacral plexuses. *Proceedings of the Royal Society of London*.

[5] Overduin S. A., d’ Avella A., Carmena J. M., Bizzi E. (2012). Microstimulation activates a handful of muscle synergies. *Neuron*.

[6] d’Avella A., Saltiel P., Bizzi E. (2003). Combinations of muscle synergies in the construction of a natural motor behavior. *Nature Neuroscience*.

[7] d’Avella A., Bizzi E. (2005). Shared and specific muscle synergies in natural motor behaviors. *Proceedings of the National Academy of Sciences*.

[8] Nazifi M. M., Yoon H. U., Beschorner K., Hur P. (2017). Shared and task-specific muscle synergies during normal walking and slipping. *Frontiers in Human Neuroscience*.

[9] Maton B., Bouisset S. (1977). The distribution of activity among the muscles of a single group during isometric contraction. *European Journal of Applied Physiology and Occupational Physiology*.

[10] Giszter S. F., Mussa-Ivaldi F. A., Bizzi E. (1993). Convergent force fields organized in the frog’s spinal cord. *Journal of Neuroscience*.

[11] Bizzi E., Mussa-Ivaldi F., Giszter S. (1991). Computations underlying the execution of movement: a biological perspective. *Science*.

[12] Chvatal S. A., Ting L. H. (2012). Voluntary and reactive recruitment of locomotor muscle synergies during perturbed walking. *The Journal of Neuroscience*.

[13] Ivanenko Y. P. (2003). Temporal components of the motor patterns expressed by the human spinal cord reflect foot kinematics. *Journal of Neurophysiology*.

[14] Mussa-Ivaldi F. Nonlinear force fields: a distributed system of control primitives for representing and learning movements.

[15] Scott S. H., Sergio L. E., Kalaska J. F. (1997). Reaching movements with similar hand paths but different arm orientations. II. Activity of individual cells in dorsal premotor cortex and parietal area 5. *Journal of Neurophysiology*.

[16] Mussa-Ivaldi F. A., Gantchev N., Gantchev G. N. (1999). Motor primitives, force-fields and the equilibrium point theory. *From Basic Motor Control to Functional Recovery*.

[17] Bizzi E., Cheung V. C. K. (2013). The neural origin of muscle synergies. *Frontiers in Computational Neuroscience*.

[18] Gandolfo F., Mussa-Ivaldi F. A., Bizzi E. (1996). Motor learning by field approximation. *Proceedings of the National Academy of Sciences of the United States of America*.

[19] Shadmehr R., Mussa-Ivaldi F. A. (1994). Adaptive representation of dynamics during learning of a motor task. *Journal of Neuroscience*.

[20] Huesler E. J., Maier M. A., Hepp-Reymond M. C. (2000). EMG activation patterns during force production in precision grip. III. Synchronisation of single motor units. *Experimental Brain Research*.

[21] Ting L. H., Mckay J. L. (2007). Neuromechanics of muscle synergies for posture and movement. *Current Opinion in Neurobiology*.

[22] Conwit R. A., Stashuk D., Tracy B., Mchugh M., Brown W. F., Metter E. J. (1999). The relationship of motor unit size, firing rate and force. *Clinical Neurophysiology*.

[23] McKay J. L., Ting L. H. (2008). Functional muscle synergies constrain force production during postural tasks. *Journal of Biomechanics*.

[24] Kargo W. J., Giszter S. F. (2008). Individual premotor drive pulses, not time-varying synergies, are the units of adjustment for limb trajectories constructed in spinal cord. *The Journal of Neuroscience*.

[25] Hart C. B., Giszter S. F. (2004). Modular premotor drives and unit bursts as primitives for frog motor behaviors. *Journal of Neuroscience*.

[26] Grinyagin I. V. (2005). Kinematic and dynamic synergies of human precision-grip movements. *Journal of Neurophysiology*.

[27] Flash T., Hochner B. (2005). Motor primitives in vertebrates and invertebrates. *Current Opinion in Neurobiology*.

[28] Tagliabue M., Ciancio A. L., Brochier T., Eskiizmirliler S., Maier M. A. (2015). Differences between kinematic synergies and muscle synergies during two-digit grasping. *Frontiers in Human Neuroscience*.

[29] Wishaw I. (2015). Arm and hand movement: current knowledge and future perspective. *Frontiers in Neurology*.

[30] Mussa-Ivaldi F. A., Solla S. A. (2004). Neural primitives for motion control. *IEEE Journal of Oceanic Engineering*.

[31] Oscari F., Finetto C., Kautz S. A., Rosati G. (2016). Changes in muscle coordination patterns induced by exposure to a viscous force field. *Journal of Neuroengineering and Rehabilitation*.

[32] Saltiel P., Wyler-Duda K., D'Avella A., Tresch M. C., Bizzi E. (2001). Muscle synergies encoded within the spinal cord: evidence from focal intraspinal NMDA iontophoresis in the frog. *Journal of Neurophysiology*.

[33] Overduin S. A., d’ Avella A., Carmena J. M., Bizzi E. (2014). Muscle synergies evoked by microstimulation are preferentially encoded during behavior. *Frontiers in Computational Neuroscience*.

[34] Overduin S. A., d'Avella A., Roh J., Carmena J. M., Bizzi E. (2015). Representation of muscle synergies in the primate brain. *Journal of Neuroscience*.

[35] Tresch M. C., Saltiel P., Bizzi E. (1999). The construction of movement by the spinal cord. *Nature Neuroscience*.

[36] Georgopoulos A. P., Kettner R. E., Schwartz A. B. (1988). Primate motor cortex and free arm movements to visual targets in three-dimensional space. II. Coding of the direction of movement by a neuronal population. *Journal of Neuroscience*.

[37] Mussa-Ivaldi F. A., Giszter S. F., Bizzi E. (1994). Linear combinations of primitives in vertebrate motor control. *Proceedings of the National Academy of Sciences of the United States of America*.

[38] Ethier C., Brizzi L., Darling W. G., Capaday C. (2006). Linear summation of cat motor cortex outputs. *Journal of Neuroscience*.

[39] Kargo W. J., Giszter S. F. (2000). Rapid correction of aimed movements by summation of force-field primitives. *Journal of Neuroscience*.

[40] Lemay M. A., Calagan J. E., Hogan N., Bizzi E. (2001). Modulation and vectorial summation of the spinalized frog’s hindlimb end-point force produced by intraspinal electrical stimulation of the cord. *IEEE Transactions on Neural Systems and Rehabilitation Engineering*.

[41] Capaday C., van Vreeswijk C. (2015). Linear summation of outputs in a balanced network model of motor cortex. *Frontiers in Computational Neuroscience*.

[42] Day S. J., Hulliger M. (2001). Experimental simulation of cat electromyogram: evidence for algebraic summation of motor-unit action-potential trains. *Journal of Neurophysiology*.

[43] Hagio S., Kouzaki M. (2015). Action direction of muscle synergies in three-dimensional force space. *Frontiers in Bioengineering and Biotechnology*.

[44] Hart C. B., Giszter S. F. (2010). A neural basis for motor primitives in the spinal cord. *Journal of Neuroscience*.

[45] Ting L. H. (2005). A limited set of muscle synergies for force control during a postural task. *Journal of Neurophysiology*.

[46] Walter J. P., Kinney A. L., Banks S. A. (2014). Muscle synergies may improve optimization prediction of knee contact forces during walking. *Journal of Biomechanical Engineering*.

[47] Aoi S., Funato T. (2016). Neuromusculoskeletal models based on the muscle synergy hypothesis for the investigation of adaptive motor control in locomotion via sensory-motor coordination. *Neuroscience Research*.

[48] Krishnamoorthy V., Latash M. L., Scholz J. P., Zatsiorsky V. M. (2003). Muscle synergies during shifts of the center of pressure by standing persons. *Experimental Brain Research*.

[49] Laczko J., Latash M. (2016). *Progress in Motor Control Theories and Translations*.

[50] Valero-Cuevas F. J., Venkadesan M., Todorov E. (2009). Structured variability of muscle activations supports the minimal intervention principle of motor control. *Journal of Neurophysiology*.

[51] Cheung V. C. K., Piron L., Agostini M., Silvoni S., Turolla A., Bizzi E. (2009). Stability of muscle synergies for voluntary actions after cortical stroke in humans. *Proceedings of the National Academy of Sciences of the United States of America*.

[52] Hashiguchi Y., Ohata K., Kitatani R. (2016). Merging and fractionation of muscle synergy indicate the recovery process in patients with hemiplegia: the first study of patients after subacute stroke. *Neural Plasticity*.

[53] Hirai H., Miyazaki F., Naritomi H. (2015). On the origin of muscle synergies: invariant balance in the co-activation of agonist and antagonist muscle pairs. *Frontiers in Bioengineering and Biotechnology*.

[54] Alkadhi H., Crelier G. R., Boendermaker S. H., Golay X., Hepp-Reymond M. C., Kollias S. S. (2002). Reproducibility of primary motor cortex somatotopy under controlled conditions. *American Journal of Neuroradiology*.

[55] Frère J., Hug F. (2012). Between-subject variability of muscle synergies during a complex motor skill. *Frontiers in Computational Neuroscience*.

[56] Overduin S. A., D'avella A., Roh J., Bizzi E. (2008). Modulation of muscle synergy recruitment in primate grasping. *Journal of Neuroscience*.

[57] Roh J., Rymer W. Z., Beer R. F. (2012). Robustness of muscle synergies underlying three-dimensional force generation at the hand in healthy humans. *Journal of Neurophysiology*.

[58] Torres-Oviedo G., Macpherson J. M., Ting L. H. (2006). Muscle synergy organization is robust across a variety of postural perturbations. *Journal of Neurophysiology*.

[59] Torres-Oviedo G., Ting L. H. (2010). Subject-specific muscle synergies in human balance control are consistent across different biomechanical contexts. *Journal of Neurophysiology*.

[60] Cheung V. C. (2005). Central and sensory contributions to the activation and organization of muscle synergies during natural motor behaviors. *Journal of Neuroscience*.

[61] Berniker M., Jarc A., Bizzi E., Tresch M. C. (2009). Simplified and effective motor control based on muscle synergies to exploit musculoskeletal dynamics. *Proceedings of the National Academy of Sciences of the United States of America*.

[62] Kargo W. J., Ramakrishnan A., Hart C. B., Rome L. C., Giszter S. F. (2010). A simple experimentally based model using proprioceptive regulation of motor primitives captures adjusted trajectory formation in spinal frogs. *Journal of Neurophysiology*.

[63] Kuppuswamy N., Harris C. M. (2014). Do muscle synergies reduce the dimensionality of behavior?. *Frontiers in Computational Neuroscience*.

[64] Kutch J. J., Valero-Cuevas F. J. (2012). Challenges and new approaches to proving the existence of muscle synergies of neural origin. *PLoS Computational Biology*.

[65] Bennett K. M., Lemon R. N. (1994). The influence of single monkey cortico-motoneuronal cells at different levels of activity in target muscles. *The Journal of Physiology*.

[66] Cherian A., Fernandes H. L., Miller L. E. (2013). Primary motor cortical discharge during force field adaptation reflects muscle-like dynamics. *Journal of Neurophysiology*.

[67] Donoghue J. P., Sanes J. N., Hatsopoulos N. G., Gaál G. (1998). Neural discharge and local field potential oscillations in primate motor cortex during voluntary movements. *Journal of Neurophysiology*.

[68] Van Antwerp K. W., Burkholder T. J., Ting L. H. (2007). Interjoint coupling effects on muscle contributions to endpoint force and acceleration in a musculoskeletal model of the cat hindlimb. *Journal of Biomechanics*.

[69] De Groote F., Jonkers I., Duysens J. (2014). Task constraints and minimization of muscle effort result in a small number of muscle synergies during gait. *Frontiers in Computational Neuroscience*.

[70] Ivanenko Y. P., Poppele R. E., Lacquaniti F. (2004). Five basic muscle activation patterns account for muscle activity during human locomotion. *The Journal of Physiology*.

[71] Hug F., Turpin N. A., Dorel S., Guével A. (2012). Smoothing of electromyographic signals can influence the number of extracted muscle synergies. *Clinical Neurophysiology*.

[72] Weiss E. J., Flanders M. (2004). Muscular and postural synergies of the human hand. *Journal of Neurophysiology*.

[73] Chiel H. J., Ting L. H., Ekeberg O., Hartmann M. J. Z. (2009). The brain in its body: motor control and sensing in a biomechanical context. *Journal of Neuroscience*.

[74] Ijspeert A. J., Crespi A., Ryczko D., Cabelguen J. M. (2007). From swimming to walking with a salamander robot driven by a spinal cord model. *Science*.

[75] Delcomyn F. (1980). Neural basis of rhythmic behavior in animals. *Science*.

[76] Grillner S. (1985). Neurobiological bases of rhythmic motor acts in vertebrates. *Science*.

[77] Inouye J. M., Valero-Cuevas F. J. (2016). Muscle synergies heavily influence the neural control of arm endpoint stiffness and energy consumption. *PLoS Computational Biology*.

[78] Prochazka A., Ellaway P. (2012). Sensory systems in the control of movement. *Comprehensive Physiology*.

[79] Prochazka A., Yakovenko S. (2007). The neuromechanical tuning hypothesis. *Progress in Brain Research*.

[80] Brown T. G. (1911). The intrinsic factors in the act of progression in the mammal. *Proceedings of the Royal Society B: Biological Sciences*.

[81] Cash S., Yuste R. (1999). Linear summation of excitatory inputs by CA1 pyramidal neurons. *Neuron*.

[82] Rathelot J. A., Strick P. L. (2009). Subdivisions of primary motor cortex based on cortico-motoneuronal cells. *Proceedings of the National Academy of Sciences of the United States of America*.

[83] Law A. J., Rivlis G., Schieber M. H. (2014). Rapid acquisition of novel interface control by small ensembles of arbitrarily selected primary motor cortex neurons. *Journal of Neurophysiology*.

[84] Nazarpour K., Barnard A., Jackson A. (2012). Flexible cortical control of task-specific muscle synergies. *Journal of Neuroscience*.

[85] Asavasopon S., Rana M., Kirages D. J. (2014). Cortical activation associated with muscle synergies of the human male pelvic floor. *The Journal of Neuroscience*.

[86] Drew T., Kalaska J., Krouchev N. (2008). Muscle synergies during locomotion in the cat: a model for motor cortex control. *The Journal of Physiology*.

[87] Rana M., Yani M. S., Asavasopon S., Fisher B. E., Kutch J. J. (2015). Brain connectivity associated with muscle synergies in humans. *Journal of Neuroscience*.

[88] Takei T., Confais J., Tomatsu S., Oya T., Seki K. (2017). Neural basis for hand muscle synergies in the primate spinal cord. *Proceedings of the National Academy of Sciences of the United States of America*.

[89] Brochier T. (2004). Patterns of muscle activity underlying object-specific grasp by the macaque monkey. *Journal of Neurophysiology*.

[90] Rouse A. G., Schieber M. H. (2016). Spatiotemporal distribution of location and object effects in primary motor cortex neurons during reach-to-grasp. *The Journal of Neuroscience*.

[91] Marder E., Bucher D. (2001). Central pattern generators and the control of rhythmic movements. *Current Biology*.

[92] Nishida K., Hagio S., Kibushi B., Moritani T., Kouzaki M. (2017). Comparison of muscle synergies for running between different foot strike patterns. *PLoS One*.

[93] Ivanenko Y. P. (2006). Spinal cord maps of spatiotemporal alpha-motoneuron activation in humans walking at different speeds. *Journal of Neurophysiology*.

[94] Tresch M. C., Cheung V. C. K., d’Avella A. (2006). Matrix factorization algorithms for the identification of muscle synergies: evaluation on simulated and experimental data sets. *Journal of Neurophysiology*.

[95] Alessandro C., Delis I., Nori F., Panzeri S., Berret B. (2013). Muscle synergies in neuroscience and robotics: from input-space to task-space perspectives. *Frontiers in Computational Neuroscience*.

[96] d'Avella A., Lacquaniti F. (2013). Control of reaching movements by muscle synergy combinations. *Frontiers in Computational Neuroscience*.

[97] Hart C. B., Giszter S. F. (2013). Distinguishing synchronous and time-varying synergies using point process interval statistics: motor primitives in frog and rat. *Frontiers in Computational Neuroscience*.

[98] Spüler M., Irastorza-Landa N., Sarasola-Sanz A., Ramos-Murguialday A. (2016). Extracting muscle synergy patterns from EMG data using autoencoders.

[99] Maier M. A., Hepp-Reymond M. C. (1995). EMG activation patterns during force production in precision grip. II. Muscular synergies in the spatial and temporal domain. *Experimental Brain Research*.

[100] Steele K. M., Tresch M. C., Perreault E. J. (2015). Consequences of biomechanically constrained tasks in the design and interpretation of synergy analyses. *Journal of Neurophysiology*.

[101] Afzal T., Iqbal K., White G., Wright A. B. (2017). A method for locomotion mode identification using muscle synergies. *IEEE Transactions on Neural Systems and Rehabilitation Engineering*.

[102] Rasool G., Iqbal K., Bouaynaya N., White G. (2016). Real-time task discrimination for myoelectric control employing task-specific muscle synergies. *IEEE Transactions on Neural Systems and Rehabilitation Engineering*.

[103] Delis I., Berret B., Pozzo T., Panzeri S. (2013). Quantitative evaluation of muscle synergy models: a single-trial task decoding approach. *Frontiers in Computational Neuroscience*.

[104] Kutch J. J., Kuo A. D., Bloch A. M., Rymer W. Z. (2008). Endpoint force fluctuations reveal flexible rather than synergistic patterns of muscle cooperation. *Journal of Neurophysiology*.

[105] Banks C. L., Pai M. M., McGuirk T. E., Fregly B. J., Patten C. (2017). Methodological choices in muscle synergy analysis impact differentiation of physiological characteristics following stroke. *Frontiers in Computational Neuroscience*.

[106] García-Cossio E., Broetz D., Birbaumer N., Ramos-Murguialday A. (2014). Cortex integrity relevance in muscle synergies in severe chronic stroke. *Frontiers in Human Neuroscience*.

[107] Roh J., Rymer W. Z., Beer R. F. (2015). Evidence for altered upper extremity muscle synergies in chronic stroke survivors with mild and moderate impairment. *Frontiers in Human Neuroscience*.

[108] Tropea P., Monaco V., Coscia M., Posteraro F., Micera S. (2013). Effects of early and intensive neuro-rehabilitative treatment on muscle synergies in acute post-stroke patients: a pilot study. *Journal of Neuroengineering and Rehabilitation*.

[109] Cheung V. C. K., Turolla A., Agostini M. (2012). Muscle synergy patterns as physiological markers of motor cortical damage. *Proceedings of the National Academy of Sciences of the United States of America*.

[110] Clark D. J., Ting L. H., Zajac F. E., Neptune R. R., Kautz S. A. (2010). Merging of healthy motor modules predicts reduced locomotor performance and muscle coordination complexity post-stroke. *Journal of Neurophysiology*.

[111] Carpinella I., Jonsdottir J., Lencioni T., Bowman T., Ferrarin M. (2016). Planar robotic rehabilitation of upper limb in post-stroke subjects: transfer of training effects to a non-trained 3D functional task. *Gait & Posture*.

[112] Dipietro L., Krebs H. I., Fasoli S. E. (2007). Changing motor synergies in chronic stroke. *Journal of Neurophysiology*.

[113] McMorland A. J. C., Runnalls K. D., Byblow W. D. (2015). A neuroanatomical framework for upper limb synergies after stroke. *Frontiers in Human Neuroscience*.

[114] Godlove J., Gulati T., Dichter B., Chang E., Ganguly K. (2016). Muscle synergies after stroke are correlated with perilesional high gamma. *Annals of Clinical and Translational Neurology*.

[115] Manickaraj N., Bisset L. M., Devanaboyina V. S. P. T., Kavanagh J. J. (2017). Chronic pain alters spatiotemporal activation patterns of forearm muscle synergies during the development of grip force. *Journal of Neurophysiology*.

[116] Brunnstrom S. (1970). *Movement Therapy in Hemiplegia: A Neurophysiological Approach*.

[117] Fugl-Meyer A. R., Jääskö L., Leyman I., Olsson S., Steglind S. (1975). The post-stroke hemiplegic patient. 1. a method for evaluation of physical performance. *Scandinavian Journal of Rehabilitation Medicine*.

[118] Cirstea M. C., Levin M. F. (2000). Compensatory strategies for reaching in stroke. *Brain*.

[119] Bowden M. G., Behrman A. L., Neptune R. R., Gregory C. M., Kautz S. A. (2013). Locomotor rehabilitation of individuals with chronic stroke: difference between responders and nonresponders. *Archives of Physical Medicine and Rehabilitation*.

[120] Jones T. A. (2017). Motor compensation and its effects on neural reorganization after stroke. *Nature Reviews Neuroscience*.

[121] Hesam-Shariati N., Trinh T., Thompson-Butel A. G., Shiner C. T., McNulty P. A. (2017). A longitudinal electromyography study of complex movements in poststroke therapy. 2: changes in coordinated muscle activation. *Frontiers in Neurology*.

[122] Chvatal S. A., Torres-Oviedo G., Safavynia S. A., Ting L. H. (2011). Common muscle synergies for control of center of mass and force in nonstepping and stepping postural behaviors. *Journal of Neurophysiology*.

[123] Yang N., An Q., Yamakawa H. Clarification of muscle synergy structure during standing-up motion of healthy young, elderly and post-stroke patients.

[124] Matsunaga N., Imai A., Kaneoka K. (2017). Comparison of muscle synergies before and after 10 minutes of running. *Journal of Physical Therapy Science*.

[125] Kristiansen M., Samani A., Madeleine P., Hansen E. A. (2016). Effects of 5 weeks of bench press training on muscle synergies. *Journal of Strength and Conditioning Research*.

[126] Shaharudin S., Zanotto D., Agrawal S. (2014). Muscle synergies of untrained subjects during 6 min maximal rowing on slides and fixed ergometer. *Journal of Sports Science & Medicine*.

[127] Grioli G., Catalano M., Silvestro E., Tono S., Bicchi A. Adaptive synergies: an approach to the design of under-actuated robotic hands.

[128] Rasool G., Iqbal K., Bouaynaya N., White G. Neural drive estimation using the hypothesis of muscle synergies and the state-constrained Kalman filter.

[129] Catalano M. G., Grioli G., Farnioli E., Serio A., Piazza C., Bicchi A. (2014). Adaptive synergies for the design and control of the Pisa/IIT SoftHand. *The International Journal of Robotics Research*.

[130] Berger D. J., d'Avella A. (2014). Effective force control by muscle synergies. *Frontiers in Computational Neuroscience*.

[131] Tresch M. C., Jarc A. (2009). The case for and against muscle synergies. *Current Opinion in Neurobiology*.

[132] Bicchi A., Gabiccini M., Santello M. (2011). Modelling natural and artificial hands with synergies. *Philosophical Transactions of the Royal Society B: Biological Sciences*.

[133] Bastian A. J. (2008). Understanding sensorimotor adaptation and learning for rehabilitation. *Current Opinion in Neurology*.

[134] Barroso F. O., Torricelli D., Bravo-Esteban E. (2016). Muscle synergies in cycling after incomplete spinal cord injury: correlation with clinical measures of motor function and spasticity. *Frontiers in Human Neuroscience*.

[135] Lunardini F., Casellato C., Bertucco M., Sanger T. D., Pedrocchi A. (2017). Children with and without dystonia share common muscle synergies while performing writing tasks. *Annals of Biomedical Engineering*.

[136] Steele K. M., Rozumalski A., Schwartz M. H. (2015). Muscle synergies and complexity of neuromuscular control during gait in cerebral palsy. *Developmental Medicine & Child Neurology*.

[137] Tang L., Chen X., Cao S., Wu D., Zhao G., Zhang X. (2017). Assessment of upper limb motor dysfunction for children with cerebral palsy based on muscle synergy analysis. *Frontiers in Human Neuroscience*.

[138] Tang L., Li F., Cao S., Zhang X., Wu D., Chen X. (2015). Muscle synergy analysis in children with cerebral palsy. *Journal of Neural Engineering*.

